# Individualized, home-based interactive training of cerebral palsy children delivered through the Internet

**DOI:** 10.1186/1471-2377-11-32

**Published:** 2011-03-09

**Authors:** Peder E Bilde, Mette Kliim-Due, Betina Rasmussen, Line Z Petersen, Tue H Petersen, Jens B Nielsen

**Affiliations:** 1 The Helene Elsass Center, Holmegårdsvej 28, 2900 Charlottenlund, Denmark; 2 Department of Neuroscience and Pharmacology, Panum Institute, University of Copenhagen, Copenhagen, Denmark; 3 Department of Exercise and Sport Sciences, University of Copenhagen, Copenhagen, Denmark

## Abstract

**Background:**

The available health resources limit the amount of therapy that may be offered to children with cerebral palsy and the amount of training in each session may be insufficient to drive the neuroplastic changes, which are necessary for functional improvements to take place. The aim of this pilot study was to provide proof of concept that individualized and supervised interactive home-based training delivered through the internet may provide an efficient way of maintaining intensive training of children with cerebral palsy over prolonged periods.

**Methods:**

9 children (aged 9-13 years) with cerebral palsy were included in the study. Motor, perceptual and cognitive abilities were evaluated before and after 20 weeks of home-based training delivered through the internet.

**Results:**

The children and their families reported great enthusiasm with the training system and all experienced subjective improvements in motor abilities and self-esteem. The children on average trained for 74 hours during a 20 week period equalling just over 30 minutes per day. Significant improvements in functional muscle strength measured as the frontal and lateral step-up and sit-to-stand tests were observed. Assessment of Motor and processing skills also showed significant increases. Endurance measured as the Bruce test showed a significant improvement, whereas there was no significant change in the 6 min walking test. Balance (Romberg) was unchanged. Visual perceptual abilities increased significantly.

**Conclusions:**

We conclude that it is feasible to deliver interactive training of children with cerebral palsy at home through the internet and thereby ensure more intensive and longer lasting training than what is normally offered to this group.

## Background

Children with cerebral palsy suffer from motor and cognitive disabilities, which usually require a multi-facetted treatment strategy over many years involving a number of different health professionals. Even with the economical resources available today in most western societies, it is doubtful whether it is possible to offer sufficiently intensive and persistent motor and cognitive training to guarantee optimal, lasting improvements for the children with current therapeutic approaches. It is now known that in order to drive neuroplastic changes in the brain much more intensive and long-lasting training is necessary than what has been assumed previously [1]. It has been estimated for quite some time as a rule of thumb that athletes need to train around 10.000 hours (which corresponds to around 5 hours pr day for 5-6 years) to become as good as possible, but although this is likely also to apply to children with brain damage and other neurological patients, it has only recently received some attention in the neurorehabilitation field. It has also now been noted that there is a great discrepancy between the intensive training used to obtain neuroplastic changes and thereby promote successful functional recovery in animal experiments and the much less intensive training offered to patients with brain damage. Cats usually make 1000-2000 steps in each session during treadmill training, whereas the average number of steps in training of human spinal cord injured subjects is only around 300 [1]. Similarly, monkeys usually train around 600 arm movements, whereas human patients train around 30 movements pr session [1]. Importantly, it is usually outside the reach of most health care systems to offer more than 1-2 30-60 min sessions of physiotherapy pr week to children with cerebral palsy. This contrasts to the 30-45 minute sessions every day, which appear to be necessary to efficiently drive neuroplastic changes [2-5].

It is thus clear that it is necessary to find cost-effective therapeutic approaches which can help children with cerebral palsy to train intensively for a sufficiently long time. We find that the only realistic way of doing this is to develop a system for home-based training where the children and their families take responsibility for the training. The advantages of this approach have been documented in several previous studies [6,7] and some kind of more or less formalised home-based training exists in many countries. However, one drawback is that home-based training is usually unsupervised. The children and their families therefore easily loose motivation, do not train sufficiently and harmful training may even take place. Also, optimizing the training individually as the child improves and obtains new functionality is difficult. With recent developments in computer-technology, the possibility of delivering and supervising training through the internet has, however, emerged. Such internet-based training has been tested in trials on patients with diabetes [8-10] and rheumatoid arthritis [11] with positive results, but to our knowledge there are no reports on a home-based training system for supervised motor and cognitive training of children (or other patients) with brain damage. Finally, studies have documented that interactive computer-games which have some element of physical and cognitive training involved are highly motivating and may help to promote some level of physical activity in children and young people for longer periods [12,13]. However, such computer-games may not be adjusted to the demands of the individual child and are therefore often too difficult for children with physical and cognitive disabilities. Except for a single case-study [14] such computer-games have therefore to our knowledge also not been implemented in the therapy of children with cerebral palsy.

The aim of the present pilot study was to provide proof of concept that an interactive home-based computer training system, which can be fully individualized and optimized to the motor and cognitive ability of the individual child at any given time during the training program, may provide an efficient way of training children with cerebral palsy for a prolonged period. The training program is delivered and optimized by a therapist through the internet and only requires a PC, a webcam and a high-speed internet connection. Our data demonstrate that it is feasible to deliver interactive training of children with cerebral palsy at home through the internet and thereby ensure more intensive and longer lasting training than what is normally offered to this group.

## Methods

### Subjects

9 children (age range: 6-13 years; mean age; 10 years and 3 month; 5 boys; Table 1) with the diagnosis spastic cerebral palsy (CP) based on medical records participated in the study. All children and their parents gave informed consent to the study which was approved by the local ethics committee of Copenhagen region (H-B-2009-017).

**Table 1:** Summary of children data at the beginning of the training.

Child	Age	Sex	MACS	GMFCS-level
1	6	M	2	I
2	9	M	2	II
3	12	F	2	I
4	12	F	1	I
5	8	M	1	I
6	12	M	2	I
7	13	F	1	I
8	12	F	2	I
9	9	M	1	I

MACS: Manual Ability Classification System. GMFCS: Gross Motor Function Classification System.

Table 1 summarizes the functional abilities of the children prior to the study. Fine and gross motor functions of the children were evaluated by the Manual Ability Classification System (MACS [15]) and the Gross Motor Function Classification System Expanded and Revised (GMFCS-E&R) [16,17], respectively.

MACS has been developed to classify how children with cerebral palsy use their hands when handling objects in daily activities. MACS is based upon self-initiated manual ability, with a particular emphasis on handling objects in an individual's personal space (the space immediately close to one's body, as distinct from objects that are not within reach).

**Level I**. Handles objects easily and successfully.

**Level II**. Handles most objects but with somewhat reduced quality and/or speed of achievement.

**Level III**. Handles objects with difficulty; needs help to prepare and/or modify activities.

**Level IV**. Handles a limited selection of easily managed objects in adapted situations.

**Level V**. Does not handle objects and has severely limited ability to perform even simple actions.

GMFCS-E&R is a classification system which is used to describe the gross motor function of children with cerebral palsy [16,17].

GMFCS-E&R is based on self-initiated movements with a focus on the ability to sit, stand and move. The classification system has 5 age groups, which each have 5 levels of function. The 5 levels are designed to ensure that the different levels reflect as much as possible genuine differences in functional ability during daily living. The main focus is therefore on functional limitations and the need of walking aids rather than the quality of movement.

**LEVEL I - **Walks without Limitations

**LEVEL II - **Walks with Limitations

**LEVEL III - **Walks Using a Hand-Held Mobility Device

**LEVEL IV - **Self-Mobility with Limitations; May Use Powered Mobility

**LEVEL V - **Transported in a Manual Wheelchair

The subsequent training was adjusted according to these different classes (see below).

### Measure tools

The motor, perceptual and cognitive abilities of the children were evaluated before and after the 20 weeks training period. All tests were carried out at the Helene Elsass center.

#### Motor abilities

AMPS (Assessment of motor and process skills) was performed according to [18]. AMPS is a computer-based, standardized, cross-cultural assessment tool for use by occupational therapists to assess the motor abilities of people in the age group 3-99 years. For the test, the patient selects two daily activities which are assessed. The quality of activity is scored from the degree of exertion, efficacy, confidence and independence in 16 individual motor and 20 processing skills. These are equivalent to the skills defined under 'Activities and Participation' in *the International Classification of Functioning, Disability and Health *(World Health Organization, WHO, 2001). Each of them are thus essential parts of the overall successful execution of a task. The motor skills are used to estimate performance when the subject moves or when the subject moves an object. The process skills are used to estimate performance when the subject a) chooses, interacts with and uses tools and materials, b) execute individual tasks and c) adjust movements when problems occur.

The performance of the child in each of the 16 motor and 20 processing skills were given a score from 1 to 4 where 1 was given for a markedly deficient performance that ***impeded ***the action progression and yielded unacceptable outcomes, 2 for an ineffective performance that ***disrupted ***or interfered with the action progression and yielded undesirable outcomes, 3 for a questionable performance that ***placed the action progression at risk ***and yielded uncertain outcomes that caused the examiner to question the adequacy of performance and 4 for a competent performance that ***supported ***the action progression and yielded good outcomes.

The individual scores from the motor and processing skills were used for a general graphical scoring in ADL units with a range from 4 to -3 for motor skills and 3 top -4 for processing skills

AHA (Assisting Hand Assessment) was performed according to [19]. It is a standardized test of hand function in children aged 18 month to 12 years with unilateral functional deficits. The AHA is conducted by observing object-related actions performed with the affected hand/arm during play with toy from the AHA test kit (suitcase). The AHA- suitcase contains a number of carefully selected toys. The toys were selected on the basis that they are normally handled with the two hands together.

The AHA play session is recorded on video in a standardized manner. The child should sit on a chair in front of and close to a table. The height of the chair should be adjusted to the height of the child, placing the table surface at a level just below the child's elbows. The examiner sits on the opposite site of the table. The camera should be placed rather high, just behind the examiner on the opposite side from the child's affected side.

The play session is to be completed in about 10-15 minutes. The session is semi structured, meaning that although the toys are predetermined, there is no specific right or wrong way in which the child is supposed to handle them or a determined order in which to present the toys to the child.

The sum of scores may vary between 22 and 88 points, where a higher score indicates a higher ability level. The scaled scores range between 0 and 100 and is a transformation of the sum score to a percentage distribution within the scale, where 100 indicates that all test items were performed with the highest scores, and 0 means that all test items were performed with the lowest point.

#### Isometric muscle strength

Isometric muscle strength in knee extensors and flexors was evaluated by an isodynamometer (HUR-Line Research, HUR-Labs, Finland). For extension the knee was kept in an angle of 120°, for flexion the knee was kept in an angle of 140°. An average of 3 trials in which the child generated maximal voluntary knee extension or flexion was calculated. Due to technical problems reliable measures were only obtained in 4 of the 9 children.

#### Functional Strength

##### Sit-to-stand, unloaded

The test was made according to [20]. The child was placed on a stool with no back rest. Hips, knees and feet were placed in 90°. Feet were kept parallel to hip. During all measurements the child held onto a 50 cm stick to prevent support on thigh or stool. The child was requested to stretch hips and knees fully during standing before sitting again. Stand-sit was considered a full cycle. Number of cycles fully carried through within 30 s were counted.

##### Sit-to-stand, loaded

As above, but the child wore a rucksack with an estimated amount of resistance (appr. 8RM). Number of possible stand-sit were counted.

##### Lateral and frontal step-up

Children in GMFCS groups I and II used a 20 cm stool, except for 3 children who used a stool 5 cm lower. For each child the same height of stool was used before and after the training period. The test was performed according to [20] During the assessment the child stood next to/in front of the stool with straight hips and knees in the supporting leg. In the start of the test the leg to be tested was placed on the stool. The child was instructed to place the supporting leg (opposite to tested leg) fully on the stool with weight on both legs, knees and hips straight before the supporting leg was placed on the floor again. Supporting leg placed on the stool and back on the floor was considered a full cycle. Number of cycles fully carried through within 30 s were counted.

In all cases the right leg was tested first, regardless of whether the child had right- or leftsided hemiplegia or diplegia

#### Balance

##### Romberg 30 seconds, eyes open

The child stood without shoes and no support on a force platform (HUR balance trainer, HUR, Helsinki, Finland). Heels were held approximately 2 cm separated with an angle of 30° between the medial sides of the feet. The arms were kept relaxed at the side of the body. The platform was placed 11/2 m from the wall which had a picture with small objects that the child could focus on. The child was instructed to focus on the picture and count silently from 1-100. The area that the center of gravity was maintained within for 90% of the time (C90 area), the average velocity of sway and the total trace length were registered.

#### Gait tests

##### Bruce treadmill test [21]

The test was performed on a treadmill (Technogym Run Med; Technogym The Wellness Company, Cesena, Italy) connected to Cardio Memory software module containing the Bruce treadmill test. The software automatically calculates a fitness rating for the child.

The test is constructed so that speed and tilt of the treadmill changes every 3 minutes beginning at 2,7 km/hour with a tilt of 10% (Table 2). The test continues until the child says stop.

**Table 2:** Bruce treadmill test.

Time (minutes)	0 min	3 min	6 min	9 min	12 min	15 min	18 min
Speed (km/h)	2,7	4,0	5,5	6,8	8,0	8,8	9,6
Tilt(%)	10	12	14	16	18	20	22

The child wore a pulse belt in order to obtain an impression of the cardiovascular challenge of the test. The child was allowed to support on the handle of the treadmill.

#### 6 minutes walking test

The child walked over-ground as fast as possible without running for 6 minutes [22]. The distance from one turning point to the next was 30 metres. The child wore shoes during the test.

Before the test began the child was instructed to walk from one turning point to the next, and *just pass *the turning point before walking back again. During the test the child was verbally encouraged ("*you are doing well*", "*very good*" etc). Approximately every minute the child was told how long time had passed. The child was allowed to take a break while standing if necessary, but not to sit down. There were no other persons or other disturbances in the room for as long as the test took place. At the end of the test the total distance and the mean speed were calculated.

#### Visual perception tests

TVPS (Test of Visual Perceptual Skills) [23].

The test consists of 7 different tests of visual perception:

1. Visuo-spatial relationships. The ability to perceive the spatial orientation of a figure. In the test the child is shown a series of designs on a page and then asked to choose the one that is different from the rest

2. Visual discrimination. The ability to visually discriminate a figure from other similar figures. The child is shown a design and asked to point to the matching design among the choices shown below.

3. Visual memory. The ability to remember a figure presented visually. The child is shown (for 5 seconds) a design on one page, the page is turned, and the child is asked to choose the same design from among the choices shown on the following page.

4. Visual sequence memory. The ability to remember a specific sequence of visual inputs. The child is shown (for 5 seconds) design sequences, the page is turned, and the child is asked to choose the matching design from the choices on the following page

5. Visual closure. The ability to recognize a not fully drawn figure. The child is shown a completed design on the page and is asked to match it to one of the incomplete patterns shown on the page

6. Visual constancy. The ability to recognize a figure although it changes shape, orientation or size. The child is asked to find one design among others on the page

7. Visual figure ground. The ability to discriminate a figure from the background. The child is asked to find one design among many within a complex background

Performance in the tests was scored by counting the number of correct answers in each test (maximally 16 in each of the 7 tests). The performance in each test was then scaled according to normative data for the child's age group and converted into percentage score for the age group. A score of 91% thus indicates that the child scored higher or equal to 91% of its age group.

The tests took place in a small office free of any auditory and visual distractions. The room was well illuminated, quiet and there was no distraction from other people in the testing room. The test took place between 10 am and 2 pm. The child sat on a chair in front of and close to a table. The examiner sat on the opposite site of the table. The test was placed between them. Item responses were made vocally (by saying the letter of the response choice) or by pointing. The responses were untimed, but the children were all encouraged to proceed as quickly as possible. Only subtest 3 (visual memory) and 4 (sequential Memory) had a time component. In these two tests the child had 5 seconds to look at the picture before the page was turned.

### Training procedure

**Figure 1:**
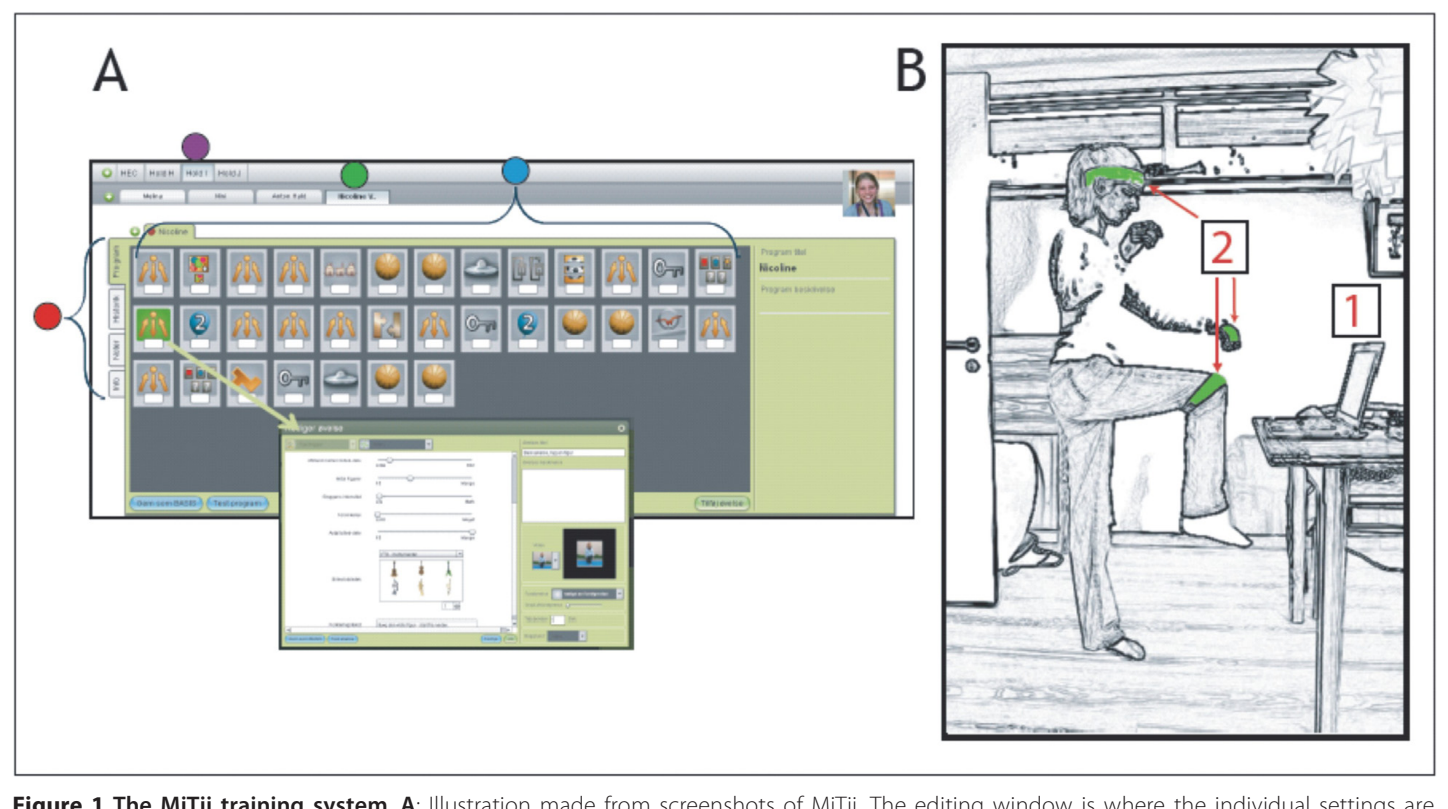
**The MiTii training system**. **A**: Illustration made from screenshots of MiTii. The editing window is where the individual settings are done and progressively modified. First step in the editing is to choose the group of patients (purple dot). Next the screen of the individual client in question is selected (green dot). Each individual training programme is made up of a sequence of exercises. The different exercises are represented by icons that can be moved around freely to accomplish the optimal individual sequence. The full program covers from 30 to 45 minutes (blue dot). A basic case record system is provided to store personal patient information (red dot). A double click on each of the icons opens up a new window (arrow). Here it is possible to adjust a range of parameters **B**: The necessary hardware comprises a computer (1) with a webcam and an Internet connection. A green elastic band (2) is placed on the person in training. The band is placed around the head, on the wrists, the knees, the elbows or wherever the therapists find it to meet the requirements.

The training of the children with CP took place in their own home during a 20 week period. The program delivered to the children ensured that they trained at least 30 minutes per day every day during the whole period. The training was delivered through the internet and consisted of a server-based interactive training-system using flash-technology. The system has been developed through a collaboration between The Helene Elsass Center, a private software development company (Head-fitted; Århus, Denmark) and the University of Copenhagen. It has now been made commercially available through collaboration between the Helene Elsass center and the Ministry of Research under the name MiTii (Move It To Improve It; MiTii developments, Charlottenlund, Denmark). The training-system is designed to combine cognitive and motor challenges in order to train cognitive, perceptual and motor abilities at the same time. It consists of a number of training modules in which the child has to analyze visual information, solve a cognitive problem (i.e. mathematical question or similar) and respond with a motor act (i.e. bend to pick up needle and blow up balloon with the right answer). The core of the system is that the computer program identifies the movements of the child from video images sampled from a simple web-camera attached to the computer. No additional computer-interface is thereby necessary - the child may control the computer-program by his or her own free movements. The motion detection is based on identification of a green band, which may be placed around the wrist, shoulder, head or leg of the child (Figure 1).

The level of difficulty may be adjusted throughout the training period by increasing the difficulty of the perceptual (e.g. increasingly complex forms have to be correctly identified), cognitive (e.g. increasingly difficult mathematical questions) or motor challenges (e.g. child has to do more repetitions or work with higher load). This adjustment was executed by therapists (PT and OT), who followed the training of the child through the internet based on feedback regarding the progress of the child. The therapists were in addition in contact through E-mail and Skype with the child and its parents on an at least weekly basis and thereby received feedback regarding the progress of the training. This had the additional effect that the child (and its parents) had the impression that they had a 'private' virtual coach who supervised the training. This was reported as one of the most motivating factors by both parents and children (see further in results).

The different modules were combined uniquely according to the specific cognitive and motor deficits of each child. The specific content of the program was decided from the baseline motor and perceptual performance of the individual child as described above.

The system thus has two clear advantages over existing training programs: It is highly adjustable to the individual needs of the child and it can be adjusted according to the progress of training of the individual child.

### Data analysis

If data were found to be normally distributed, measurements before and after the 20 week training period were compared using a paired Student's t test, otherwise Wilcoxon signed rank test was used. All data are given as population mean +/- 1 SEM.

## Results

### Training duration

On average the 9 children trained on 119 +/- 8.9 days (range: 111-138 days) out of the scheduled 140 days (corresponding to an average of 85% (range: 79.3-98.5%)). The children on average trained 36.6 +/- 3.8 minutes per day reaching a total average of 73.6 +/- 8.0 hours (range: 62-82 hours). This is a little above the 70 hours of training, which was the aim of the project (at least 30 minutes every day in the 140 day period = 70 hours). 6 of the children managed to train more than this. In total the children trained more than 30 minutes on 783 days out of the total 1260 training days; corresponding to 62%.

### Subjective reports

All children and their families reported great satisfaction with the training system, although the children found it very hard - and at times boring - to do the requested 30 minutes of training every day for all 20 weeks. All families experienced difficulties persuading the children to do the training in periods. On the other hand many families also experienced that their child showed great enthusiasm for the training and many of them invited friends to be present while training. The families reported that they found that the most motivating factor was the contact with the therapists through e-mail, which made them feel that they were not left alone with the training, but that each child had a 'virtual coach'. The game-like design of the training system was reported to be one of the initial motivating factors for most of the children, but following weeks of training this subsided. Instead, as the children experienced that the training system improved their functional abilities, a desire to improve their abilities became the dominant motivating factor. All families reported that the trained child showed signs of improved mobility in daily life, increased muscle strength, increased endurance and improvement in a number of skills in daily life. All families indicated that the single most important effect of the training system, as they experienced it, was that the child had gained much more self-confidence and dared to take on much more challenges than before.

### Motor and process abilities

**Figure 2:**
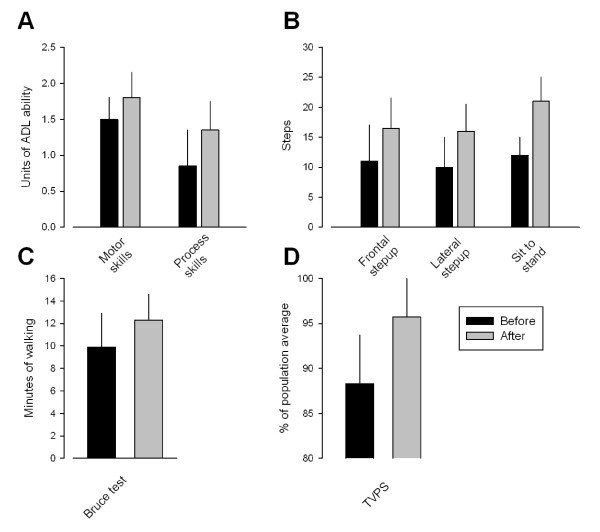
**Changes in AMPS, muscle strength, gait and TVPS following the 20 week training program**. A-C: Black and grey columns give group averages before and after the training, respectively. Each vertical line is one SEM. In A the ordinate gives the ADL ability in the motor skills test (left columns) and processing skills test (right columns). In B the ordinate gives the number of steps that the children could make on average in the frontal step-up (left columns), lateral step-up (middle columns) and sit to stand (right columns) tests. In C the ordinate is the number of minutes that the children were able to walk on average. In D the ordinate is the total score in the 7 TVPS tests as a percentage of the average score for an age-matched healthy population. Black columns are values before training and grey columns are values after training. vertical lines give 1 SEM.

The children as a group showed a significant increase in motor and process skills as tested by AMPS (Figure 2A). The score for motor skills increased from an average of 1.49 +/- 0.37 to 1.84 +/- 0.37 (p < 0.001), whereas the processing skills increased from 0.87 +/- 0.40 to 1.29 +/- 0.38 (p < 0.05).

There were no significant changes in the AHA score in the 6 children in whom this score was obtained before and after the training (average 65% vs 61% before and after the training; p = 0.4).

### Strength tests

The isometric strength tests of knee flexors and extensors were only performed in 4 children for technical reasons. Although some increase was observed in knee extensor strength in all 4 children, this did not reach statistical significance for the group (p = 0.2). Knee flexor strength showed no or only little change (p = 0.8).

The frontal and lateral step-up tests were performed in all 9 children (Figure 2B). A statistically significant increase of the number of steps was observed for both tests (11.6 to 17 steps; p < 0.001 for the frontal step up test; 10.2 to 16.4 steps for the lateral step up test; p < 0.01). A statistically significant increase was also observed for the sit-to-stand test (from 11.8 to 20.4; p < 0.01; Figure 2B).

### Balance test

The Romberg test showed no statistically significant changes.

### Gait tests

The Bruce test was performed in all 9 children, whereas the 6 min walking test was possible in only 4 of the children. Following the 20 week training period the children walked on average 12 minutes on the treadmill as compared to 9.8 minutes prior to the training, which was statistically significant (p < 0.05; Figure 2C). No statistically significant changes were observed for the 6 min walking test.

### Perceptual tests

Although a tendency for an increased number of correct responses was observed for 5 of the 7 visual perception tests (Figure 2D), only the figure ground test showed a significant change after the training for the group of children (from 8.5 to 12.75; p < 0.05). The combined score showed a significant improvement for the group of children (change from 88.3 +/- 5.4% before to 95.7 +/- 5.9% after the training; p < 0.01).

## Discussion

The purpose of this study was to report our initial experiences with a novel interactive computer-training system delivered through the internet (Mitii). Our main aim was to determine whether the training system is an effective way of encouraging, motivating and maintaining more intensive and long lasting training than what is usually offered to children with cerebral palsy. The children trained on average around 74 hours during the 20 week training period, corresponding to an average of around 30 minutes per day, which was the goal that was set for the children before the training period. Although no precise estimate exists of the amount of physiotherapy and occupational therapy normally offered to children with CP, we believe that the amount of training that the children performed far exceeds what is normally possible to offer from the official health care system even in wealthy countries. We also believe that the intensity of the training that was achieved exceeds what is normally achieved in conventional physiotherapy. It has been reported that patients perform around 30 arm movements in a conventional physiotherapy session [1], whereas the children in our study performed around 135 reaching movements per session. Since the children in our study trained every day, which is normally not the case in conventional physiotherapy, we believe that both the intensity and the volume of training achieved were much higher than what is easily achieved with other means. The training system thus offers an important step towards achieving the number of repetition of exercises which appears necessary to drive neuroplastic changes [1]. The significant improvement that we observed in the AMPS and functional strength measures is well in line with this.

It should be pointed out that since the study was designed as an open study in only 9 children without any control group, it is not possible to make any firm conclusions as to whether this way of training is more efficient in promoting functional benefits than conventional training. Importantly, except for the AMPS and TVPS tests, the study was also not controlled for the effect of increased age of the children during the training period and it therefore cannot be excluded that some of the improvements were simply caused by the higher age of the children at the end of the training period. Finally, it should be pointed out that only children in GMFCS classification groups I and II were included in the study and that the study therefore does not necessarily apply to children with more severely affected motor function. However, we would like to emphasise that despite these limitations the study shows that this way of training is feasible and that it may help children with CP train at a much higher intensity and for a much longer time than what is possible to assure with the conventional training offered by the health care system. Whether the training is sufficiently efficient to elicit functional benefits that are larger than what might be obtained by other approaches will have to await future controlled studies in larger populations of children.

An important issue in relation to this type of home-based training is to what an extent the training was accepted by the children and their families and made a part of daily life. The fact that all children except two managed to train almost every day for the required 30 minute period testifies that this was indeed the case. However, this did not happen automatically. Most of the children found that it was 'hard work' to do the training every day and all the families experienced problems persuading the children to do the training. Although the training system in many ways resembles a computer game this was apparently not sufficient to maintain interest for the whole 20 week period. Repetition of already well known training modules is probably the main reason for this. Later during the training period two other motivating factors were clearly of larger importance: 1) Most families found that the knowledge of having close contact through e-mail and Skype to a therapist, who could follow the progress of the training on a daily basis, was the most important motivating factor. The families and the children did not feel that they were left alone with the training, although it took place in their own home, but rather felt that they had a 'personal virtual coach', who followed them and inspired them to continue the training. 2) Most families also reported that a very strong motivating factor for their children later in the training period was that they experienced that the training system improved their abilities and made them capable of doing things that they were not able to do before. This may not reflect an objective improvement in functional abilities caused directly by the training, since many of the skills that were experienced as being improved by the children and their families were not part of the training system. Much of the improvements that the families experienced (such as improvements in self-esteem) is therefore more likely related to psychological factors when participating in a training project such as this rather than to a direct physical consequence of the training per se. Nevertheless, it was clear that this had the positive effect of helping the children to maintain motivation and continue training.

It was somewhat surprising that no improvement was observed in the balance tests, since most of the training modules required a certain amount of balance training. A likely explanation is that the tests were simply not sufficiently sensitive to show significant changes with the relatively small number of children in the study. In several of the tests valid data were only obtained in less than 6 children. Another possibility is that the training was not sufficiently intense and challenging to improve the measured parameters. The children were not required to directly concentrate on their balance, but only had to control balance as part of other tasks. It is possible that more specific balance training is necessary in order to obtain significant changes. Such training modules are now being implemented in the training system.

The improvements in functional muscle strength were to be expected given the high number of repetition of the exact movements that were assessed in the tests. Especially the incremental loading of the limbs during the training period will have helped to efficiently improve the functional muscle strength of the children. The small improvement in the Bruce test, which reflects to some extent an improvement in aerobic capacity [21], is in line with several studies, which have shown that computer-based indoor training involves only little aerobic exercise as compared to out-door activities, but nevertheless may lead to small but significant improvements in aerobic capacity [12,13]. The training modules did require sufficient aerobic challenge to make the children relatively exhausted at the end of the daily training program and many of them reported to be out of breath regularly during the training. It should be mentioned in this relation that children with CP are generally in a worse physical shape than most other children [24] and that the relatively light aerobic challenge in this type of computer-based training may be sufficient to make a significant difference in a long training period as this. The improvement that we observed was also well within the range of improvements observed in previous training studies in children with cerebral palsy [25,26]. However, it should also be mentioned that the Bruce test is a rather indirect test of aerobic capacity and is heavily influenced by the motivation of the child. It is therefore not impossible that non-specific psychological factors such as changes in the self-esteem of the child and its willingness to take on challenges have influenced this test result.

The training also significantly improved the visual ability of the children. Before the training their performance on the tests was 11-12% below average for their age group, whereas after the training their performance was only 4-5% below age matched healthy children. This is of interest since this suggests that the visuo-cogntive motor abilities trained by the computer-system may be transferred to every-day tasks. The finding also emphasizes that the training system may be efficient at training several different capabilities (muscle strength, endurance, motor skills, visual and cognitive abilities) at the same time.

## Conclusions

In conclusion we have found that it is feasible to deliver training at home through the internet and thereby ensure more intensive and longer lasting training than what is normally offered to this group of children.

## Competing interests

The authors declare that they have no competing interests.

## Authors' contributions

PEB and JBN conceived the study. All authors participated in the design and planning of the project. MKD, BR, LZP and THP trained all children and made the evaluations. PEB and JBN analysed all data and wrote the first draft of the manuscript. All authors read and approved the final manuscript

## Pre-publication history

The pre-publication history for this paper can be accessed here:

http://www.biomedcentral.com/1471-2377/11/32/prepub
